# Sarin: a never-ending story

**DOI:** 10.1007/s00204-022-03417-9

**Published:** 2022-11-28

**Authors:** Hermann M. Bolt

**Affiliations:** Leibniz Research Centre for Working Environment and Human Factors at TU Dortmund (IfADo), Ardeystr. 67, 44579 Dortmund, Germany

Archives of Toxicology have a history of 70 years in publishing research reports on acetylcholine esterase inhibitors (Vogel 1953). Historical milestones were early publications on delayed neuropathy (Johnson 1977, Lotti and Johnson 1978, Willams et al. 1984) by organophosphorus nerve agents, as well as studies on pharmacokinetics and pharmacodynamics of oxime antidotes, such as the “Hagedorn oxime” HI6 that was most effective in animals poisoned with the chemical warfare agents sarin, VX or soman (Klimmek and Eyer 1986; Eyer et al 1988).

Mass poisonings were reported in Japan, both caused by sarin, in Matsumoto City in 1994 (Morita et al 1995) and in the Tokyo underground railway in 1995 (Suzuki et al 1995). In both cases, analyses of sarin in the environment such as air, water, and materials left by the terrorist(s), or the decomposed compounds in the soil were helpful to understand the causative material of the incident. Especially, the quantitation of sarin metabolites proved as a useful tool of exposure monitoring (Nakajima et al 1998).

Reports on “Gulf War Illness” led to speculations on possible associations between toxic exposures. Depleted uranium as a cause has been ruled out (Bolt 2022), leaving exposure to aerosolized organophosphate compounds (pesticides and sarin nerve agent) as the more likely cause(s) of Gulf War Illness (Parrish and Haley 2021).

In the meantime, a prevalence case–control study drawn from the U.S. Military Health Survey’s National Population sample (Haley et al 2022) has supported such a causal relationship. The investigation started with pre-stated hypothesis of an association of Gulf War Illness with a gene–environment interaction of the paraoxonase-1 (PON1) Q192R polymorphism and low-level nerve agent exposure. The PON1 gene contains a common polymorphism in codon 192 of either the glutamine (Q) isoenzyme or the arginine. QQ homozygous individuals produce only the Q isoenzyme, which effectively hydrolyzes nerve agents like sarin. By contrast, RR homozygotes produce only the R isoenzyme, which is relatively ineffective against nerve agents. A prevalence sample of 508 Gulf War Illness cases and 508 non-paired controls was drawn from the 8,020 participants in the U.S. Military Health Survey, as representative sample of military veterans who served during the Gulf War. The PON1 Q192R genotype was measured by real-time polymerase chain reaction, and the serum Q and R isoenzyme activity levels were measured with PON1-specific substrates. Low-level nerve agent exposure was estimated by survey questions on having heard nerve agent alarms during deployment. The gene–environment interaction of the Q192R genotype and hearing alarms was strongly associated with Gulf War Illness on both the multiplicative [prevalence odds ratio (POR) of the interaction = 3:41; 95% confidence interval (CI) 1.20, 9.72] and additive (synergy index = 4:71; 95% CI 1.82, 12.19) scales, adjusted for measured confounders. The Q192R genotype and the alarms variable were independent (adjusted POR in the controls = 1:18; 95% CI 0.81, 1.73; *p* = 0:35). The adjusted relative excess risk due to interaction was 7.69 (95% CI 2.71, 19.13).

In total, these data support the idea of a causal relationship of low-level organophosphorus compounds, especially sarin, with symptoms of Gulf War Illness. In the lay press (Anonymous 2022), speculations were advanced of a connection with the bombing of Sadam Hussein’s cache of chemical weapons in January 1991 around the cities of Muthanna and Fallujah that had released sarin.

Given the complexity of systemic toxic actions of sarin, especially those affecting the brain (Abou-Donia 2016) and the lack of effective antidotes against CNS damage (Sakurada and Ohta 2020), research into the toxicity of sarin will continue.
